# Supplementary material for: Application of 18F-FDG Positron Emission Tomography/Magnetic Resonance in Evaluation of Oropharyngeal Carcinoma
Source: Diagnostics (Basel). 2025 Apr 24;15(9):1081. doi: 10.3390/diagnostics15091081 (PMC12071206; doi:10.3390/diagnostics15091081)
Supplement: Supplementary file 1 [file diagnostics-15-01081-s001.zip › diagnostics-3553628-supplementary.pdf]

	Enhanced-CT	Enhanced-MR	PET/MR
T stage			
T1	2	2	2
T2	7	8	7
T3	1	0	1
T4	3	3	3
N stage			
N0	3	4	3
N1	4	3	6
N2	5	5	3
N3	1	1	1
Maximal tumor diameter (cm) (*P*=0.907)			
Average (n=13)	2.76±0.92	2.84±0.77	2.69±0.54

Supplementary Table S1. TN staging and maximal tumor diameter of all patients with oropharyngeal carcinoma before treatments.

	True positive (n)	False positive (n)	True negative (n)	False negative (n)
Enhanced CT	7	4	213	7
Enhanced MR	9	2	215	5
PET/MR	11	2	215	3

Supplementary Table S2. The positive and negative cervical lymph nodes findings by enhanced CT, MR and PET/MR imaging compared with pathological results.

	Pathological TN stage				*p*
	T1		T2		
Parameters of tumor					
ADC (min)	758.50±335.88		566.40±241.55		0.423
ADC (max)	1098.00±339.41		1314.20±237.67		0.368
ADC (mean)	909.50±289.20		942.20±179.53		0.857
SUV (max)	15.67±14.85		23.58±6.64		0.337
SUV (peak)	9.75±8.85		16.02±3.59		0.202
SUV (mean)	10.09±9.87		14.30±3.57		0.397
TLG	21.17±13.59		93.68±85.97		0.312
MTV	2.76±1.35		6.72±5.42		0.377
	N0	N1	N2	N3	*p*
Parameters of lymph node					
ADC (min)	830.00	561.33±231.56	573.50±0.71	367.00	0.495
ADC (max)	1270.00	1169.67±157.18	1180±145.66	1071.00	0.833
ADC (mean)	1000.00	927.33±107.45	847±24.04	770.00	0.384
SUV (max)	5.44	16.45±4.28	9.97±2.48	16.04	0.209
SUV (peak)	3.14	9.01±2.59	6.83±0.00	10.08	0.238
SUV (mean)	3.08	10.26±3.08	6.10±1.15	8.67	0.255
TLG	4.47	11.95±6.35	87.74±102.50	68.06	0.579
MTV	1.45	1.24±0.65	16.58±19.73	7.85	0.610

Supplementary Table S3. Relationship between PET/MR parameters (ADC/SUV/TLG/MTV) and pathological TN stage.

Parameters of tumor	p16		
	Negative	Positive	*p*
ADC (min)	596.67±207.91	601.85±241.70	0.968
ADC (max)	1275.00±269.28	1170.00±170.38	0.414
ADC (mean)	925.00±200.79	905.57±166.25	0.852
SUV (max)	25.07±9.66	19.03±7.44	0.229
SUV (peak)	17.70±7.61	13.53±5.79	0.260
SUV (mean)	15.33±5.79	12.10±4.75	0.291
TLG	157.64±137.11	72.41±60.82	0.164
MTV	8.86±6.46	6.05±4.12	0.361

Supplementary Table S4. Relationship between PET/MR parameters (ADC, SUV, TLG, MTV) and p16

Parameters of tumor	Tumor differentiation			
	Poorly	Moderately	Well	*p*
ADC (min)	668.50±255.13	504.33±158.32	697.67±258.18	0.370
ADC (max)	1186.25±126.74	1143.33±166.32	1413.00±342.00	0.215
ADC (mean)	971.25±142.44	824.67±115.58	1018.67±271.22	0.230
SUV (max)	15.35±9.23	23.44±6.04	27.21±10.21	0.175
SUV (peak)	9.92±6.20	16.94±3.77	19.86±7.58	0.083
SUV (mean)	9.57±5.75	14.64±3.51	16.88±6.02	0.158
TLG	39.63±52.03	136.01±89.51	149.40±173.67	0.283
MTV	3.88±3.82	9.13±5.02	8.40±6.93	0.309

Supplementary Table S5. Relationship between PET/MR parameters (ADC/SUV/TLG/MTV) and tumor differentiation.
